# From the Editor's desk

**DOI:** 10.1111/irv.12311

**Published:** 2015-04-23

**Authors:** Jonathan S Nguyen-Van-Tam

**Affiliations:** Professor of Health Protection, University of Nottingham School of MedicineNottingham, NG5 1PB, UK

It is now just over 1 year since I took over the Editorship of Influenza and Other Respiratory Viruses (IRV). In that time, there have been many changes to the journal and, of course, in the wider world of respiratory viruses. I'd like to begin by thanking my predecessor and the journal's Founding Editor, Dr. Alan Hampson. Alan set up, nurtured and grew IRV into a vibrant and successful publication before officially handing over the reins in early 2014. He remains on the team as one of our Senior Editors. Indeed my task as Editor-in-Chief would be impossible without the fantastic and dedicated team of Senior Editors, Associate Editors and professional staff at Wiley, who support me, and all of whom deserve many personal thanks.

The challenges posed by respiratory viruses remain as important as ever for global public health, and clinical practice. In the aftermath of the 2009 pandemic, governments and public health agencies are wrestling with ‘pandemic fatigue’, austerity programmes (that limit the appetite and ability to invest in pandemic preparedness), and a false impression among some politicians that since the 2009 pandemic was rather mild and ‘not much to write home about’, pandemic preparedness is in fact ‘no big deal’. In amongst this mix are real issues pertaining to the ongoing controversy about the effectiveness of antiviral drugs,^1^–^3^; and the fact that current vaccine manufacturing platforms can only offer commercial quantities of pandemic vaccine some four to 6 months after a novel virus has emerged, thus substantially reducing the overall public health benefits, even though vaccines themselves are effective.^4^,^5^

Most public health agencies and individual experts, consider the potential pandemic threat posed by influenza A(H5N1) to be undiminished. But in addition, influenza A(H7N9) is now, if anything, considered a higher potential risk.^6^ Recent human cases of influenza A(H10N8),^7^ and A(H5N6),^8^ further remind us that the risk assessment landscape for influenza is constantly evolving and, in turn, this demands constant vigilance from the public health and scientific communities.

If one shifts the focus away from influenza, the ongoing MERS-CoV outbreak in the Middle East, is also of substantial concern, because despite its likely introduction into humans via close contact with dromedary camels,^9^ nosocomial transmission appears to be a central concern,^10^,^11^ case-fatality is high, household transmission is also described,^12^ and there are currently no vaccines or specific therapies available. Finally enterovirus D68 seems to be emerging as a potentially important respiratory pathogen in children.^13^

Seasonal influenza too, should not be overlooked as an ongoing problem. In the northern hemisphere winter of 2014–15 we have experienced substantial influenza A(H3N2) activity. Unfortunately this has coincided with a poorly matched H3N2 vaccine component that has resulted in low effectiveness against the circulating H3N2 strains in the community.^14^ This has recently been linked to excess winter mortality across Europe in the population aged 65 years and over,^15^ illustrating that we need more broadly protective vaccines, especially in the elderly.

From the journal's perspective, to be in the best possible position to respond to these emerging and sometimes fast-moving threats, we have made some recent changes that enable us to publish findings with minimal delay. For papers of significant importance, we can offer a rapid peer-review scheme, which should enable us to make a decision on a manuscript within 14 days (or less). We have also improved our arrangements for all accepted articles (whether fast-tracked or not). Following acceptance, we can now publish all articles, as unedited manuscripts, online within 1 week of acceptance.

We have also reconsidered the arrangements for review articles that we publish. We recognise that Systematic Reviews and Meta-analyses have become a central part of evidence synthesis in modern science and medicine. With this has come the setting of standards for the conduct of such work: the Preferred Reporting Items for Systematic Reviews and Meta-Analyses (PRISMA) guidelines;^16^,^17^ and we now require any systematic reviews that we accept to conform to these principles. However, we shall continue to accept Expert Commentary articles alongside as these continue to be relevant and useful to our readers.

Hopefully these changes, along with our now well-established Open Access format will maintain the journal as a vibrant publication that is relevant, and highly accessible, to scientists and clinicians working in the field of respiratory virus infection for the foreseeable future.

## Rapid peer-review procedures

Authors have the option to request rapid peer-review. Papers considered for rapid peer-review will need to be of immediate relevance, interest, or importance to scientists, clinicians, public health practitioners or policy makers, usually in relation to a current or evolving event related to respiratory virus activity. In general, papers that report data more than 6 months old are unlikely to be considered eligible. If your paper qualifies for rapid peer-review, the journal will aim to have your paper turned round within 14 days. There is no additional charge for authors with rapid peer-review.

Step #1 - Authors will need to contact the Editorial Office irv.eo@wiley.com, at least 1 week before online submission with an abstract; list of authors; please ensure a PRISMA checklist is completed if your paper is a Systematic Review article; details of research funding and disclosures of potential conflicts of interest should also be included.
Step #2 - The Editors will confirm whether your paper will qualify for rapid peer-review. The decision is final and non-negotiable.
Step #3 - Submit the paper via the ScholarOne system.

## Accepted articles

Accepted Articles have been accepted for publication and undergone full peer review but have not been through the copyediting, typesetting, pagination and proofreading process. Accepted Articles are published online a few days after final acceptance, appear in PDF format only, are given a Digital Object Identifier (DOI), which allows them to be cited and tracked, and are indexed by PubMed.
